# Classifying Neuronal Cell Types Based on Shared Electrophysiological Information from Humans and Mice

**DOI:** 10.1007/s12021-024-09675-5

**Published:** 2024-07-08

**Authors:** Ofek Ophir, Orit Shefi, Ofir Lindenbaum

**Affiliations:** 1 Faculty of Engineering, Bar-Ilan University, Ramat-Gan, Israel; 2 The Leslie & Susan Goldschmied (Gonda) Multidisciplinary Brain Research Center, Bar-Ilan University, Ramat-Gan, Israel

**Keywords:** Electrophysiology, Cell-type classification, Neuronal types, Domain adaptation, Sparse neural networks, Interpretable neural networks

## Abstract

The brain is an intricate system that controls a variety of functions. It consists of a vast number of cells that exhibit diverse characteristics. To understand brain function in health and disease, it is crucial to classify neurons accurately. Recent advancements in machine learning have provided a way to classify neurons based on their electrophysiological activity. This paper presents a deep-learning framework that classifies neurons solely on this basis. The framework uses data from the Allen Cell Types database, which contains a survey of biological features derived from single-cell recordings from mice and humans. The shared information from both sources is used to classify neurons into their broad types with the help of a joint model. An accurate domain-adaptive model, integrating electrophysiological data from both mice and humans, is implemented. Furthermore, data from mouse neurons, which also includes labels of transgenic mouse lines, is further classified into subtypes using an interpretable neural network model. The framework provides state-of-the-art results in terms of accuracy and precision while also providing explanations for the predictions.

**Supplementary Information:**

The online version contains supplementary material available at 10.1007/s12021-024-09675-5.

## Introduction

The classification of neurons, which are the basic units of the nervous system, has been a persistent challenge in neuroscience since the publication of Ramon y Cajal’s ’Histology of the Nervous System of Man and Vertebrates’ (Cajal, 1995). Classification of neurons is crucial for understanding brain function in both healthy and diseased states. It allows for consistent identification of neurons across different laboratories and experimental conditions (Zeng & Sanes, 2017). At the most fundamental level, cells can be categorized into non-neuronal cells and neurons. While neurons share the fundamental cellular structures of other cells, they are specialized in their ability to communicate through electrochemical signaling (Bean, 2007).

Neurons are cells that can produce and transmit electrical signals known as Action Potentials (AP). The electrical potential of neurons can be measured using micro-electrodes placed across their membrane. Normally, the electrical potential of neurons is negative, ranging from -40 to -90 millivolts. This potential difference is created by the concentration of ions inside and outside the cell. When a stimulus is applied to the cell, the membrane potential increases. If the stimulus is strong enough, it can cause the membrane potential to reach a threshold, which triggers the neuron to transmit an AP (Carter & Bean, 2009; Baranauskas, 2007).

Neurons can be divided into two categories: excitatory and inhibitory neurons (Melzer & Monyer, 2020). The main difference between the two types is that excitatory neurons release neurotransmitters, typically glutamic acid, that stimulate the postsynaptic neuron to produce an action potential. Conversely, inhibitory neurons release neurotransmitters, such as gamma-aminobutyric acid (GABA), which prevent the firing of an action potential. Although inhibitory interneurons make up only 10-20% of the total neural population in the cortex, they play a crucial role in sensation, movement, and cognition (Swanson & Maffei, 2019).

Physiologically, neurons consist of dendrites that serve as the input, the soma (cell body) that contains the nucleus, and the axon that serves as the output. Excitatory neurons are generally spiny in structure, with a long apical dendrite. They exhibit less variability in their electrophysiological characteristics, which makes it challenging to differentiate between subtypes of excitatory cell types using electrophysiological features alone. Inhibitory neurons, on the other hand, are typically either aspiny or sparsely spiny, with a more compact dendritic structure. They have a more significant variance in electrophysiological properties and tend to spike faster (Kawaguchi, 1997; Strübing et al., 1995). Neurons can also be classified based on their neurotransmitter, GABAergic neurons which are inhibitory cells, and Glutamatergic neurons, which are habitually excitatory and brain-area specific. Notably, it is more accessible to measure electrophysiological than morphological or genetic features (Zeng & Sanes, 2017); hence, we focus on neuronal classification exclusively relying on electrophysiology.

**Fig. 1:**
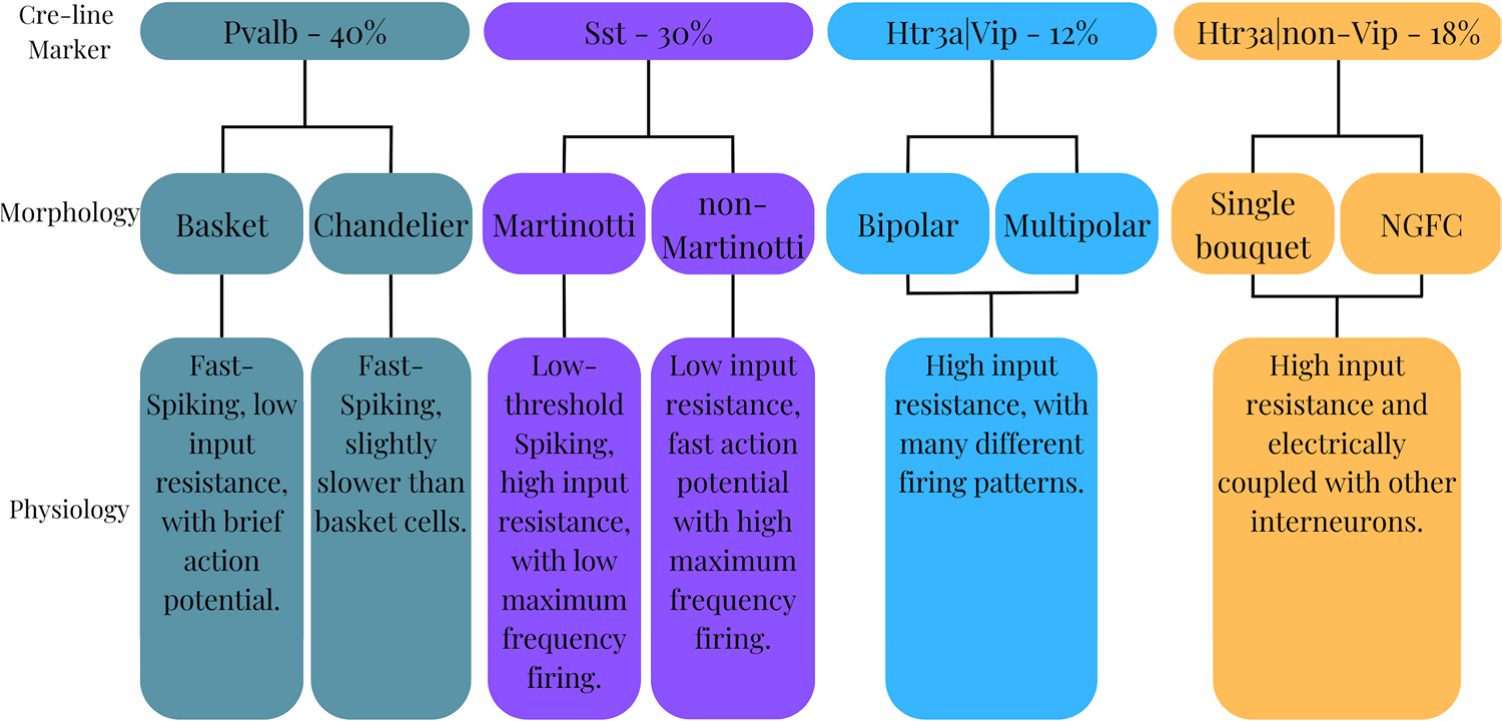
Taxonomy of GABAergic neurons in the neocortex in terms of marker genes, morphology and physiology, based on Tremblay et al. (2016)

Many neuroscientists also consider GABAergic neurons as belonging to one of the four subclasses based on the expression of specific principal gene markers (Tremblay et al., 2016); these include *Pvalb* (parvalbumin) positive, *Vip* (vasoactive intestinal peptide) positive, *Sst* (somatostatin) positive, *Htr3a* (5-hydroxytryptamine receptor) positive but *Vip* negative. These subclasses of GABAergic interneurons account for most neurons in specific brain regions, as demonstrated in Fig. 1. The classes are expressed in a non-overlapping manner, meaning that each neuron belongs to one class in a monovalent fashion, with distinct cell types accompanied by different physiological properties.

In 2019, the Allen Cell Types Database (ACTD) became publicly available (For Brain Science AI, 2015; Gouwens et al., 2019). Thanks to recent advancements in computing capabilities and the rapid development of machine and deep learning methods, the field of neuronal cell-type classification has made significant progress. The ACTD identified 17 electrophysiological neuronal types, four of which were classified as excitatory subtypes, and 13 as inhibitory subtypes. The 13 inhibitory subtypes were further categorized into the four inhibitory interneuron types based on genetic tags: *Vip*, *Ndnf*, *Sst*, and *Pvalb*. Additionally, the researchers identified 38 morphological and 46 morpho-electric neuronal types, all categorized using current clamp electrophysiological recordings. These groups underwent a dimensionality reduction process using algorithms such as principal component analysis (Abdi & Williams, 2010) and t-distributed stochastic neighbor embedding (Van der Maaten, 2008), and were then clustered using a Gaussian mixture model (Reynolds, 2009).

In their study, Ghaderi et al. (2018) developed a semi-supervised method using the Automated Clustering Tool for Deconvolution (ACTD) to classify neurons into three types: excitatory pyramidal cells (Pyr), *Pvalb* interneurons, and *Sst* interneurons from layer 2/3 of the mouse primary visual cortex. The authors achieved accuracies of 91.59 ± 1.69, 97.47 ± 0.67, and 89.06 ± 1.99 for *Pvalb*, Pyr, and *Sst* neurons, respectively. This resulted in an overall accuracy of 92.67 ± 0.54% for the classification of the three neuron types.

Seo and Lee (2019) used machine learning to predict transgenic markers of neurons using electrophysiological recordings. The work evaluated three different methods, namely: Random Forest (RF) (Ho, 1995), Least Absolute Shrinkage and Selection Operator (Tibshirani, 1996), and Fully Connected Neural Network (FCNN) (Rosenblatt, 1958). The three models performed similarly in predicting transgenic markers for excitatory neurons (*Ctgf*, *Cux2* &*Slc17*, *Nr5a1* &*Scnn1a*, *Ntsr1*, *Rbp4*, and *Rorb*) with an accuracy range of 28.57-46.93%. They also predicted transgenic markers for inhibitory neurons (*Chrna2*, *Gad2*, *Htr3a*$+|$*Vip*−, *Ndnf*, *Nkx2*, *Pvalb*, *Sst*, *Vip* &*Chat*) with an accuracy range of 59.03-73.49%.

More recently, Rodríguez-Collado and Rueda (2021) revealed a circular ordered taxonomy by transforming the first two principal components of the electrophysiological features PCA. They validated the proposed taxonomy with several machine learning models: linear discriminant analysis (Balakrishnama & Ganapathiraju, 1998), RF, gradient boosted decision tree (Chen & Guestrin, 2016; Natekin & Knoll, 2013), support vector machine (Cortes & Vapnik, 1995), and FCNN ensemble (Zhou & Chen, 2002). These models were able to discriminate the different neuron types (4 types of inhibitory neurons - *Pvalb*, *Htr3a*$+|$*Vip*−, *Sst*, *Vip*, as well as Glutamatergic excitatory cells) using electrophysiological features with accuracy ranging between 66.1-75.2% for the raw data, and 72.0-80.3% accuracy for a subset of the data that has been cleaned using anomaly detectors.

The studies mentioned so far have only relied on neuronal data collected from mice samples, as access to human neuronal data is limited. Even though mouse and human neuronal cell types and functionality are similar (Wong et al., 2023), electrophysiological differences between neurons from the two different species still exist, such that human pyramidal neurons have a higher action potential threshold voltage, a lower input resistance, and larger dendritic arbors (Mihaljević et al., 2021).

One can overcome the limitation of scarce neuronal data availability using a machine-learning model that is trained on data from both humans and mice. However, this approach may result in overfitting to the most abundant domain, leading to performance gaps on data from other domains (Novak et al., 2018; Farahani et al., 2021). This problem is known as the domain shift problem, and it can be addressed by using tools from the domain adaptation field (Zhou et al., 2022). Neural networks are known as "black boxes" due to their complexity, which makes it challenging to understand their predictions (Gunning et al., 2019). In the field of biomedicine, it is essential to interpret the model’s predictions as practitioners need to validate and trust them. Therefore, model interpretability is crucial. Additionally, due to their complexity, neural networks are often referred to as "black boxes", making it difficult to interpret their predictions Gunning et al. (2019). In the field of biomedicine, model interpretability is crucial as practitioners need to trust and validate the predictions made by the model.

This paper presents a machine-learning framework that addresses the challenge of predicting neuronal cell types in two defined tasks. In the first task, the framework suggests enriching the scarce human neuronal data using data from mice. The same neural network is used to classify broad neuronal types (excitatory vs. inhibitory) in both human and mouse samples. The mouse source data (which is easier to obtain) is used to learn a distribution over the human target data by embedding mutual information of the two domain distributions. This improves the model’s results of the target data, i.e., human neuronal data. The second task involves classifying neurons into five neuronal subtypes, including Excitatory Glutamatergic cells and the different subclasses of inhibitory GABAergic cells (*Pvalb*, *Htr3a*$+|$*Vip*−, *Sst*, and *Vip*) in an explainable way. An inherently interpretable model is used to predict the five neuronal subclasses in a clear and understandable manner. Our algorithms achieve state-of-the-art evaluation metrics in classifying mouse neuronal types and add the ability to classify human neuronal types in a joint classification approach.

The code and data used for this research is publicly available at^1^ and^2^ respectively.

## Methods

### Data

The Mouse Neuronal Data contains recordings of whole-cell current clamp from identified fluorescent Cre-positive neurons or nearby Cre-negative neurons in acute brain slices derived from adult mice. To identify cells from mice, transgenic mouse lines harboring fluorescent reporters are used, along with drivers that allow for enrichment of cell classes based on marker genes. On the other hand, Human Neuronal Data is obtained from donated ex vivo brain tissues that are analyzed from neurosurgical and postmortem sources. This data is less abundant than data from mice and is challenging to obtain. It is available thanks to the generosity of tissue donors. The ACTD (Allen Cell Types Database) has electrophysiological recordings from 1920 mice and 413 human cells. Each whole-cell current clamp recording responds to a stimulation recorded at 200 KHz (before 2016) or 50 KHz (after 2016).

The dataset contains four stimulating conditions that evoke AP responses from the neurons. The first type, noise stimulation, comprises noise pulses of square current injections. The second type, ramp stimulation, involves gradually increasing the intensity of square current injections at a rate slower than the neuron’s time constant. The third stimulation type, long square stimulation, employs square pulses of extended duration to induce a response from the neuron. Finally, the fourth stimulation type, short square stimulation, delivers brief square pulses designed to elicit a single AP from the neuron. This focused stimulation allows a simple protocol to produce an AP.

The classification of neurons into their broad types in humans and mice, and the classification of mouse cells into their specific neuronal subclasses, relies on analyzing 41 electrophysiological tabular features, though neuronal morphology-related features are attached to some neurons in the dataset, morphological features were not used for classification. All 41 tabular features are solely based on electrophysiological behavior, this is done so that the method presented can be used in clinical real-time applications, where morphological features are lacking. Nevertheless, the morphology of the neuron is still expressed via its electrophysiological features, since the morphology affects the propagation of the electrophysiological signal in the neuronal branching trees (Ofer et al., 2017). These features are extracted from APs captured within various stimulations, such as AP width and height, and AP threshold, along with features related to AP trains, such as firing speed. Each whole-cell current clamp recording is solely based on membrane potential measurements and responds to a stimulation recorded at 200 KHz (before 2016) or 50 KHz (after 2016). Recordings are performed using a range of stimulus protocols, including short pulses (3 ms current injections used to find the action potential threshold within 10 pA), long steps (1s current injections from -110pA to rheobase +160pA, in 20pA increments), slow ramps (ramp of 25pA per 1 second, terminated after a series of action potentials are acquired), and naturalistic noise (pink noise scaled to three amplitudes, 0.75, 1, and 1.5 times rheobase.) to characterize the intrinsic properties of the neurons. For more information on the electrophysiological description of all features or stimulations used, please refer to the Allen Cell Electrophysiology Overview documentation^3^.

The Allen Institute has identified the dendritic morphology of each neuron by categorizing it as either aspiny, sparsely spiny or spiny. This was done by observing the slides of the neuron’s dendrites under a microscope at 20X or 63X magnification. These different dendritic types can be roughly classified as interneurons (aspiny and sparsely spiny) and pyramidal or spiny stellate neurons (spiny).

Aspiny dendrites are characterized by the absence of spiny protrusions, lack of a pronounced apical dendrite and/or axon that emerged from the soma or dendrite at odd angles, and had extensive local branching. Sparsely spiny dendrites are defined by the presence of infrequent to moderately frequent spiny protrusions (approximately one spine per 10 microns), lack of a pronounced apical dendrite and/or an axon that emerged from the soma or dendrite at odd angles, and had extensive local branching, and/or projected up to layer 1.

Spiny dendrites are defined by the presence of frequent spiny protrusions (approximately one spine per 1-2 microns), an axon that descended perpendicularly down to the white matter with sparse, proximal branching occurring at right angles to the primary axonal branch and/or a pronounced primary, apical dendrite (For Brain Science AI, 2015).

**Fig. 2:**
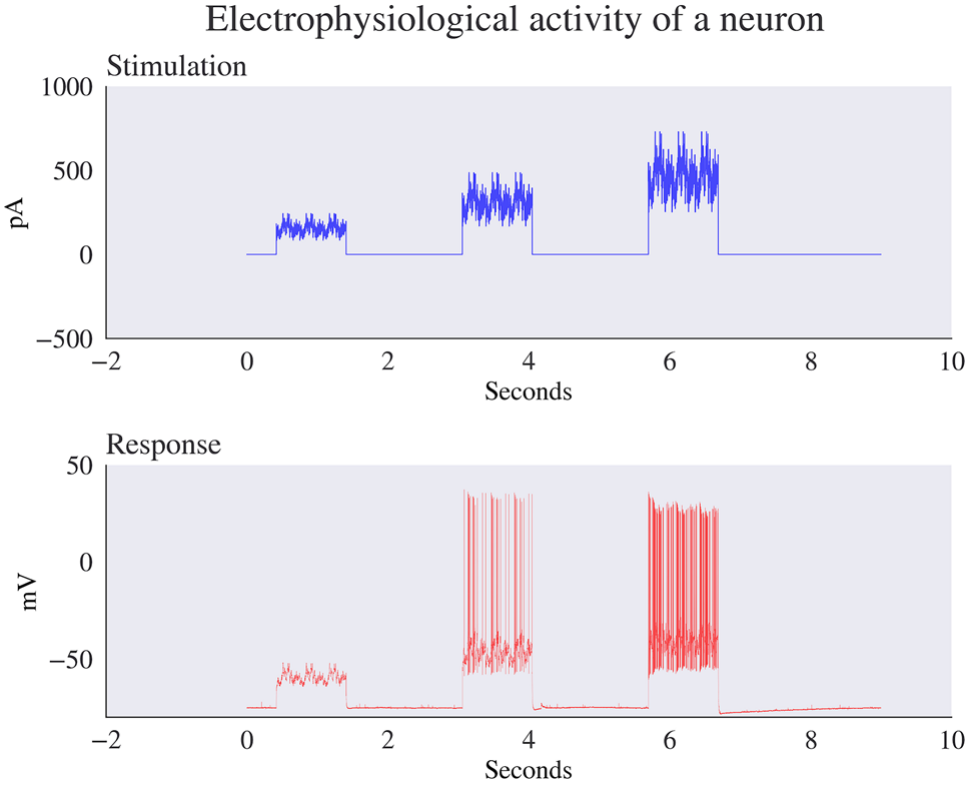
Top: Stimulation of noise pulses with square current injections scaled to three amplitudes, 0.75, 1, and 1.5 multiplied by the rheobase (the minimum current required to depolarize a nerve given an infinite duration of stimulation) with a coefficient of variation (CV) equal to 0.2. Bottom: the cell’s response to the stimulation

The dataset contains four types of stimulations: (1) noise stimulations that involve square current injections with noise pulses, (2) ramp stimulations that are square current injections with increasing intensity at a rate slower than the neuron’s time constant, (3) long square stimulations that are square pulses of duration allowing the neuron to reach a steady state, and (4) short square stimulations that are brief enough to elicit a single action potential. Below is an example of an electrophysiological response of a neuron to a noise-type stimulation in Fig. 2.

Neurons can be classified into two categories: GABAergic and Glutamatergic. GABAergic neurons are further divided into four subclasses based on their expressed Cre lines, which are *Pvalb* (Parvalbumin) positive, *Vip* (Vasoactive intestinal peptide) positive, *Sst* (Somatostin) positive, and 5-hydroxytryptamine receptor 3A (*Htr3a*) positive *Vip* negative. On the other hand, Glutamatergic neurons are classified based on their laminar locations and the location to which they project their axons, as highlighted in Tremblay et al. (2016).

**Fig. 3:**
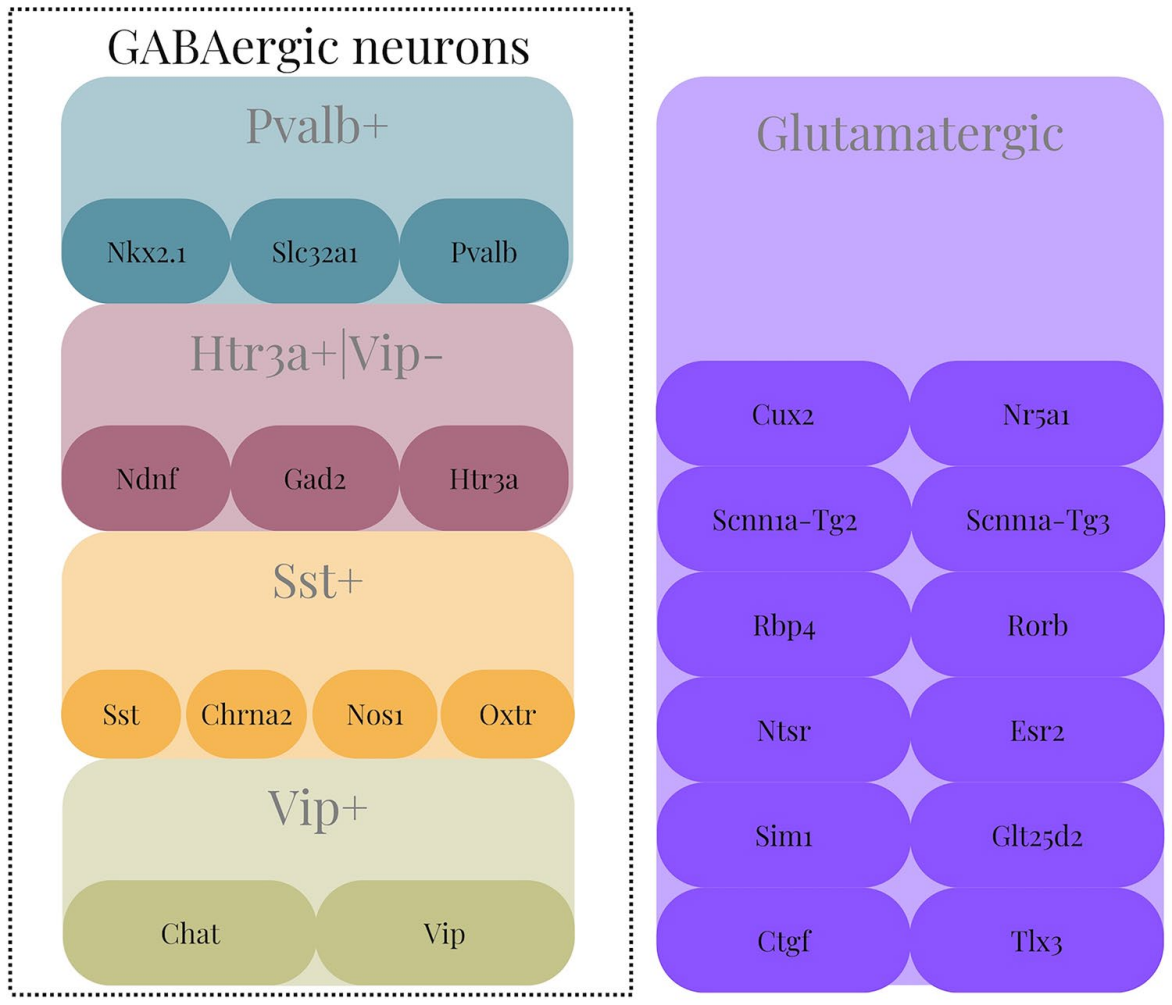
Cre-lines composing the defined subclasses, based on Rodríguez-Collado and Rueda (2021)

Using the ACTD, researchers have defined five transcriptomic-electrophysiological subclasses, including four major GABAergic subclasses and one Glutamatergic subclass. The subclasses are specified in Fig. 3, as per Rodríguez-Collado and Rueda (2021); Tremblay et al. (2016).

**Fig. 4:**
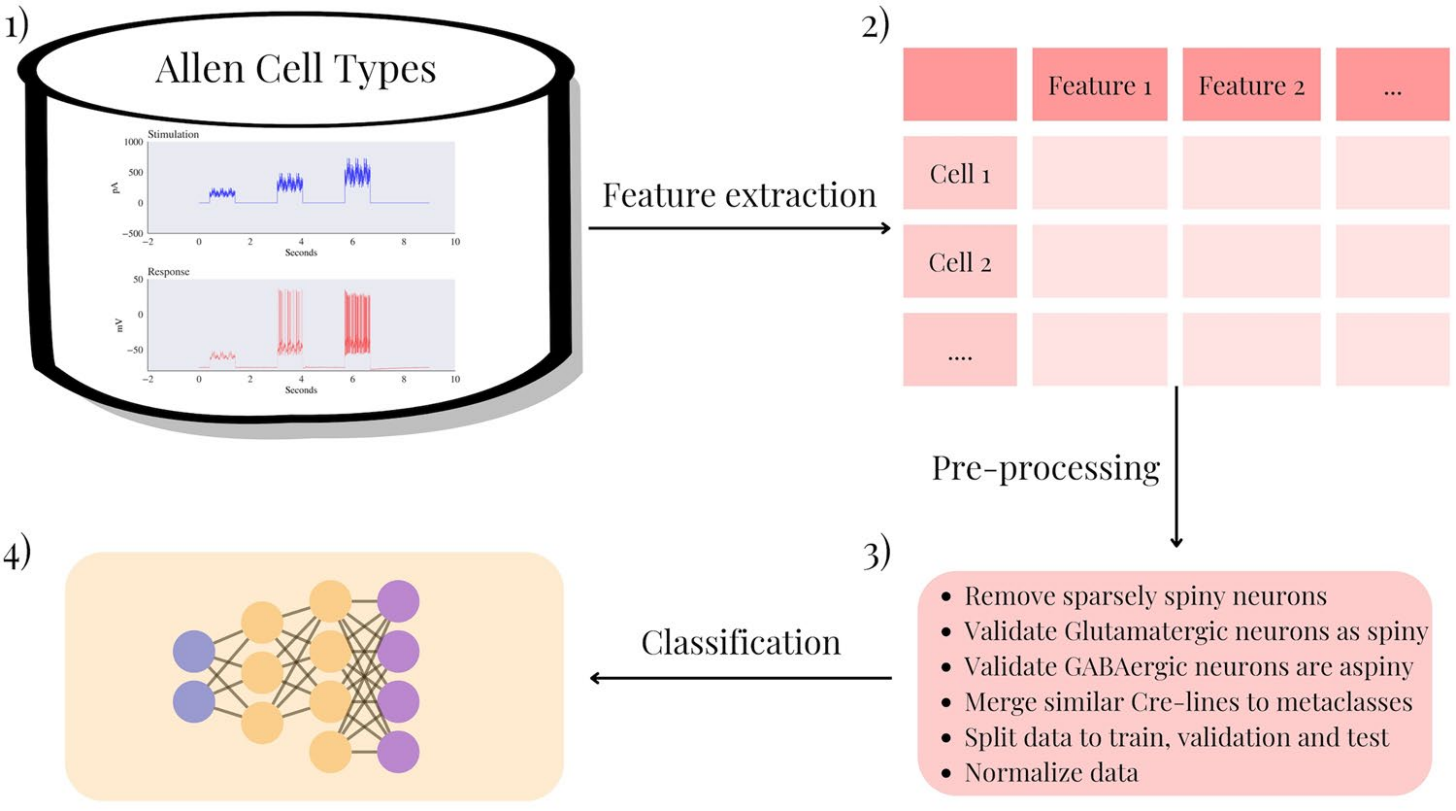
Electrophysiological features pipeline. Data is first obtained from the ACTD; then, features are extracted into a tabular format as described in Section 1 of the supplementary material. Similar transgenic lines are merged, and data is split into train, validation, and test. The data is then normalized and classified as described in Fig. 4

**Fig. 5:**
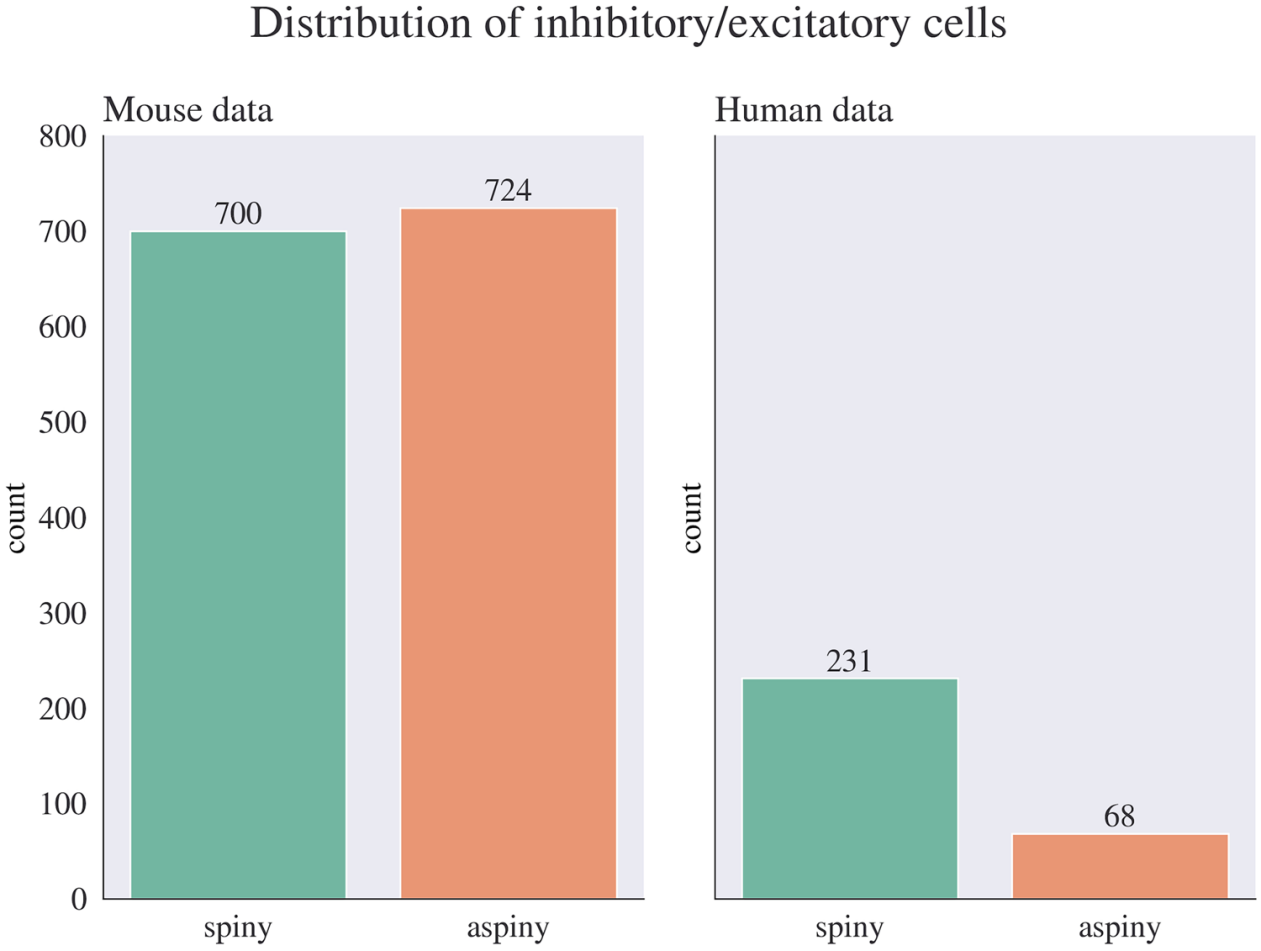
Distribution of dendrite type in mouse vs. human data

After the preprocessing stage, which is visualized in Fig. 4, we are left with 1424 mouse samples and 299 human samples for further analysis. These samples will be used to train, validate, and test our classification models. Among the mouse samples, 700 samples are classified as Glutamatergic and correspond to spiny neurons, while the other 724 samples are classified as GABAergic and pertain to aspiny neurons. In the case of human samples, we have 231 spiny neurons and 68 aspiny neurons available for analysis. The distribution of inhibitory and excitatory cells in humans and mice is illustrated in Fig. 5.

**Fig. 6:**
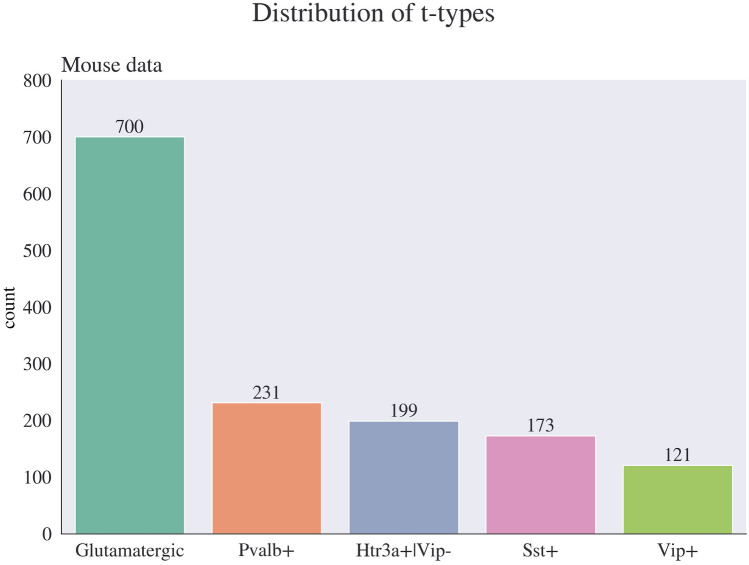
Distribution of dendrite type in mouse vs. human data

When we examine the Cre-line subclasses present in the GABAergic mouse samples, we see that there are four types. 231 neurons belong to the Pvalb subclass, 199 neurons exhibit Htr3a positivity with Vip negativity, 173 neurons fall into the Sst subclass, and 121 neurons are identified as Vip positive. You can see the graphical representation of this data in Fig. 6.

The extracted tabular AP features can be found in the supplementary material.

### Classification Models

Artificial neural networks (ANN) are a type of machine learning models inspired by biological neural networks. ANNs rely on matrix multiplications followed by nonlinear activation functions to learn complex relations between input and output. ANNs are comprised of artificial neurons that are connected through edges. These edges typically have a weight value that can adjust the strength of the signal at that connection, and the weights are ’learned’ through an optimizer such as Stochastic Gradient Descent (SGD) (Ruder, 2016).

Over the last decade, numerous neural network architectures have been developed for diverse applications (Liu et al., 2017). In this paper, we focus on fully connected neural networks, also referred to as multi-layer perceptron (MLP), or just a ’neural network’ (NN) (Krogh, 2008). We also use a new type of NN designed for tabular data, namely the Locally SParse Interpretable Network - LSPIN (Yang et al., 2022).

We focus on two classification tasks that rely solely on electrophysiological features. In the first task, we use a joint model to classify neurons from humans and mice according to their dendrite type (spiny vs. aspiny). In the second task, we classify neurons from mouse samples into their respective cell classes based on marker genes (multi-label classification). These include *Pvalb*, *Sst*, *Vip*, *Htr3a*$+|$*Vip*−, and Glutamatergic neurons. For the first task, we introduce a domain adaptation component to handle measurements from humans and mice using a joint model based on mutual information from the two domains. For the second task, we use a NN with a sample-specific feature selection mechanism, namely LSPIN, to reduce model overfitting in low-sample-size data and obtain an interpretable model.

#### Domain-adaptive Classification

In this section, we aim to design a model that can classify neurons from human samples to neuronal types, yet this is difficult due to the shortage of data samples for human neurons. Human neuronal samples are scarce and difficult to obtain. To overcome this issue, we use both mouse and human samples to establish a shared distribution of similar characteristics from both domains. The underlying assumption is rooted in the biological similarity between mouse and human neurons, both originating from mammalian brain tissues. We effectively classify neuronal samples by utilizing common information within samples from both species. However, conventional ANNs may exhibit sub-optimal performance and underperform in such a scenario due to the domain shift arising from the distinct distributions of mouse and human neuronal data. We overcome this limitation by introducing an adversarial domain adaptation scheme, namely DANN Ganin et al. (2016), designed to mitigate the influence of this domain shift. This scheme aligns the distributions of mouse and human neuronal data, enhancing the model’s ability to classify human neuronal samples accurately.

We consider $X \in \mathbb {R}^{D}$ the input space, and $Y \in \{0, 1\}$ the output space, where 0 is an excitatory cell, and 1 is an inhibitory cell. We define *S* to be the source distribution over $X \times Y$, and $D_S$ to be the marginal distribution such that $S = \{(\varvec{x}_i, y_i)\}_{i=1}^n \sim D_S$. We define *T* to be the target distribution over $X \times Y$, and $D_T$ to be the marginal distribution such that $T = \{(\varvec{x}_i, y_i)\}_{i={n+1}}^{N} \sim D_T$. Where *n* is the number of source samples, and *N* is the total number of samples. We aim to define a classifier $\eta : X \rightarrow {} Y$ to which the target risk function in Eq. (1) is low while maintaining a low source risk:

1 $$\begin{aligned} R_{D_T}(n) = \underset{\{\varvec{x}, y\} \sim D_T}{Pr} (\eta (\varvec{x}) \ne y). \end{aligned}$$

Since there may be a shift between $D_S$ and $D_T$, training a naive model based on Eq. (1) can be biased towards the more abundant domain $D_S$. To alleviate such bias, Ganin et al. (2016) introduced a technique called DANN that combines representation learning (i.e., deep feature learning) and unsupervised domain adaptation in an end-to-end training process. DANN jointly optimizes two adversarial losses, minimizing the loss of a label classifier and maximizing the loss of a domain classifier. Training both losses can be considered a form of adversarial neural network regularization. On the one hand, the network needs to classify the data into the correct labels. On the other hand, the predictions made by the network must be based on features that cannot discriminate between the source domain and the target domain. In our setting, mouse cells are considered the source distribution and are more abundant, and the human cells serve as the target distribution.

**Fig. 7:**
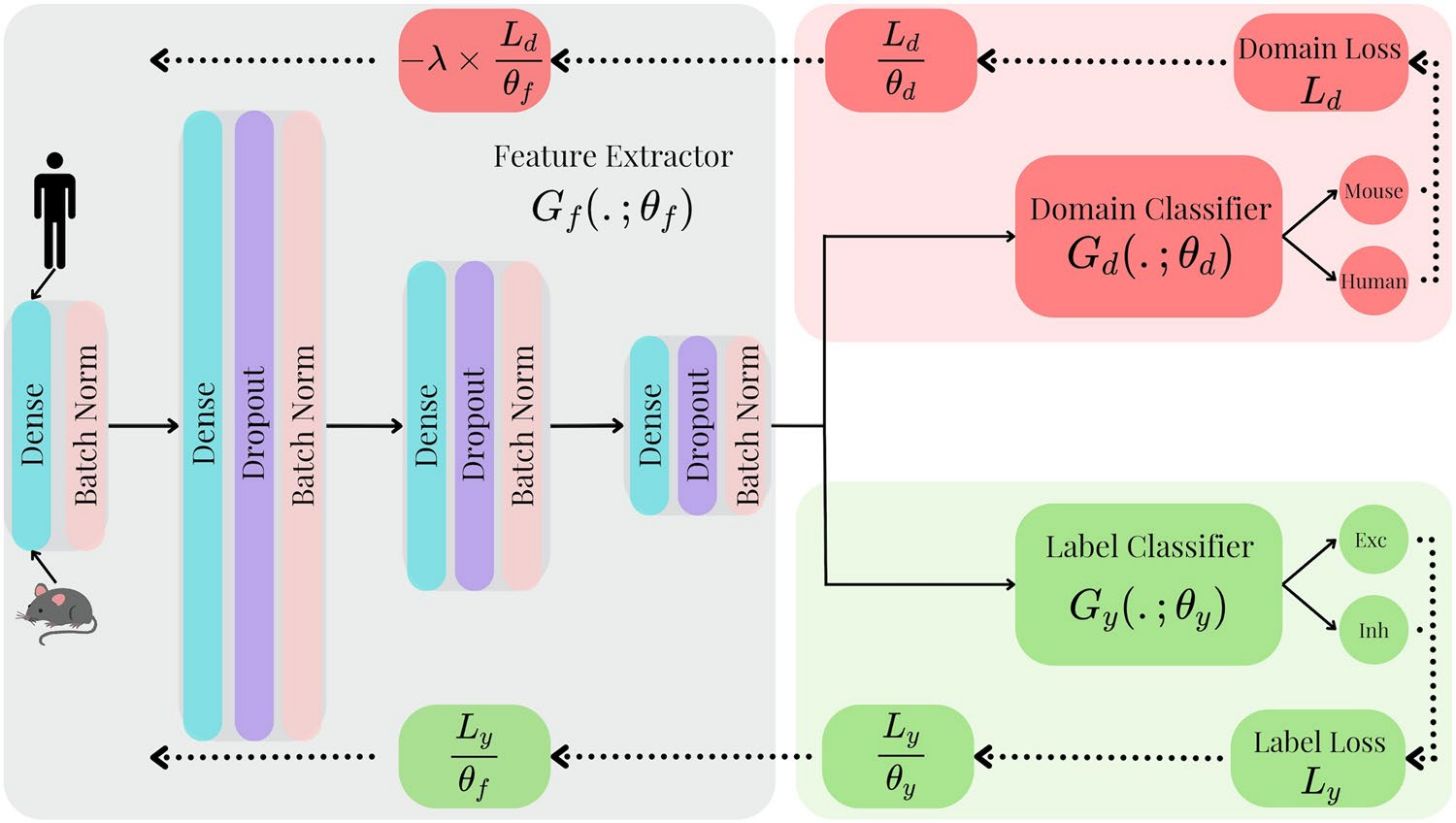
The architecture of the DANN. During forward propagation (solid-line), input data may come from humans or mice. The feature extractor (gray) with weights $\theta _f$ outputs to both the domain classifier (red) with weights $\theta _d$ and label classifier (greed) with weights $\theta _y$. During backpropagation (dotted line), the domain gradient is multiplied by a negative constant. In contrast, the label gradient remains positive. The model cannot differentiate between human and mouse samples but is nonetheless forced to classify cell types for both domains. When optimized, the feature extractor embeds common information from human and mouse samples such that the domain classifier cannot distinguish samples from the two domains. At that stage, the label classifier predicts the cell types of input samples from the embeddings of common information in human and mouse samples in a domain-invariant manner

The prediction loss and domain loss are respectively defined as:

$$\begin{aligned} L_y^i(\theta _f, \theta _y) = L_y(G_y(G_f(\varvec{x}_i;\theta _f);\theta _y),y_i),\\ L_d^i(\theta _f, \theta _d) = L_d(G_d(G_f(\varvec{x}_i;\theta _f);\theta _d),d_i), \end{aligned}$$

where $\theta _f, \theta _y, \theta _d$ are the parameters of the feature extractor, label classifier, and domain classifier, respectively, $G_f, G_y, G_d$ are the function outputs of the feature extractor, label classifier and domain classifier, respectively, and $d_i$ is the domain label of sample *i* as illustrated in Fig. 7.

Overall, training the model consists of optimizing Eq. (2):

2 $$\begin{aligned} E(\theta _f, \theta _y, \theta _d) = \frac{1}{N} \sum _{n=1}^{N} L_y^i(\theta _f, \theta _y) - \frac{\lambda }{N} \sum _{n=1}^{N} L_d^i(\theta _f, \theta _d), \end{aligned}$$

by finding the saddle point $\hat{\theta }_f, \hat{\theta }_y, \hat{\theta }_d$ such that:

3 $$\begin{aligned} (\hat{\theta }_f, \hat{\theta }_y) = \arg \min _{\theta _f, \theta _y} E(\theta _f, \theta _y, \hat{\theta }_d), \\ \hat{\theta }_d = \arg \max _{\theta _d} E(\hat{\theta _f}, \hat{\theta _y}, \theta _d). \nonumber \end{aligned}$$

To optimize over Eq. (3), we can use gradient descent, which relies on the following update rules:

$$\begin{aligned} \theta _f \leftarrow {} \theta _f - \mu (\frac{\partial L_y^i}{\partial \theta _f} - \lambda \frac{\partial L_d^i}{\partial \theta _f}), \\ \theta _y \leftarrow {} \theta _y - \mu \frac{\partial L_y^i}{\partial \theta _y}, \\ \theta _d \leftarrow {} \theta _d - \mu \lambda \frac{\partial L_d^i}{\partial \theta _d}. \end{aligned}$$

Where $\mu$ is the learning rate.

Using the aforementioned NN architecture, domain adaptation is achieved by forcing the prediction based on features that cannot discriminate between mouse and human samples. Final classification decisions are made using discriminative features that are domain (organism) invariant. We assume that a good representation for cross-domain transfer is one for which an algorithm cannot identify between the two domains (Farahani et al., 2021; Rozner et al., 2023).

#### Multi-label Classification Using Locally Sparse Networks

Collecting whole-cell current clamp recordings is labor intensive; for instance, the ACTD contains only 1920 mice and 413 human cells. The low number of samples makes it challenging to train an over-parametrized NN while avoiding overfitting. To overcome this limitation, we adopt a recently proposed method for fitting ANN models to low sample size data to address this obstacle. Specifically, the method is designed to deal with the problem of low sample size data for tabular heterogeneous biological data such as whole-cell current clamp recordings of neurons in various brain areas in mice. In this section, we use Locally SParse Interpretable Network - LSPIN (Yang et al., 2022), an intrinsically interpretable model with the benefit of showing the features it relied on during inference. We use LSPIN to predict five distinct neuronal types, four subclasses from GABAergic neurons, and one class of Glutamatergic neurons for a total of five subclasses. We achieve state-of-the-art results using the proposed method, surpassing other machine learning models and works in this field.

LSPIN is a locally sparse neural network in which the local sparsity is learned to identify the subset of the most relevant features for each sample. LSPIN includes two neural networks trained in tandem. The first is the gating network that predicts the sample-specific sparsity patterns, and the second is the prediction network that classifies the neuron type. By forcing the model to select a subset of the most informative features for each sample, we can reduce overfitting in low sample size data. Another benefit of this model is that by predicting the most informative features locally, we obtain an interpretation of the predictions.

Given labeled observations $\{\varvec{x}^{(i)}, y^{(i)}\}_{i=1}^N$, where $\varvec{x}^{(i)} \in \mathbb {R}^D$, and $x_d^{(i)}$ represents the d^th^ feature of the i^th^ sample, and $y^{(i)}$ is the label of the i^th^ sample. We want to learn a global prediction function $\varvec{f_\theta }$, and a set of parameters $\{\mu _d^{(i)}\}_{d=1, i=1}^{D, n}$ such that $\mu _d^{(i)}$ depict the behavior of the local stochastic gates $z_d^{(i)} \in [0, 1]$ that sparsify (for each instance *i*) the set of features that propagate into in the prediction model $\varvec{f_\theta }$. Stochastic gates (Yamada et al., 2020) are continuously relaxed Bernoulli variables highly effective for the sparsification of ANNs. They were previously used for several applications, including feature selection (Shaham et al., 2022; Jana et al., 2021) and sparse Canonical Correlation Analysis (Lindenbaum et al., 2021).

**Fig. 8:**
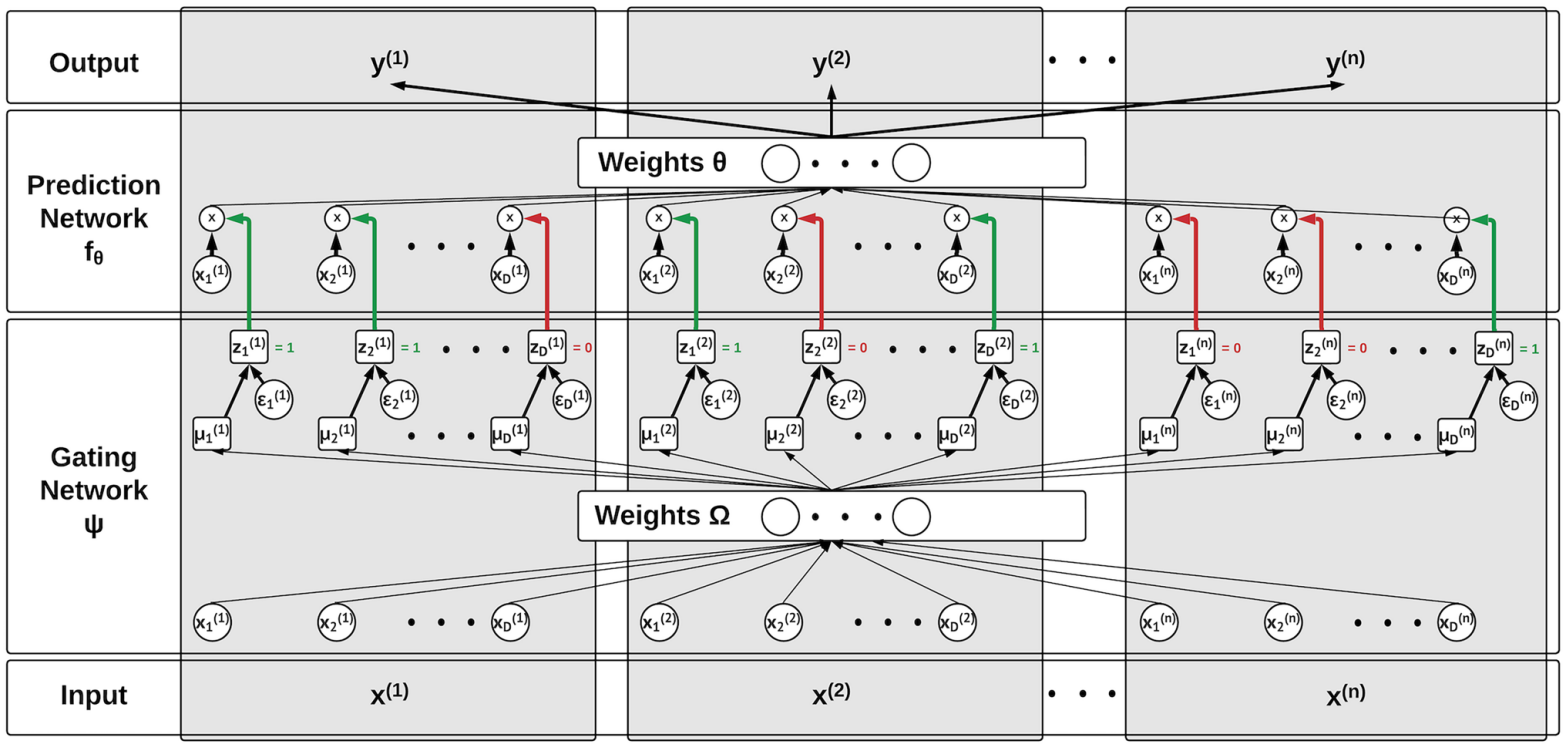
The architecture of LSPIN. The data ${\varvec{x}^{(i)} = [x_1^{(i)}, x_2^{(i)},..., x_D^{(i)}]}_{i=1}^n$ is fed simultaneously to a gating network $\varvec{\Psi }$ and to a prediction network $\varvec{f_\theta }$. The gating model learns to sparsify the set of features propagating to the prediction model, leading to sample-specific (local) sparsification. Therefore, it can handle extreme cases of low-sample-size data and lead to interpretable predictions.

Each stochastic gate (for feature *d* and sample *i*) is defined based on the threshold function in Eq. (4):

4 $$\begin{aligned} z_d^{(i)} = max(0, min(1, 0.5 + \mu _d^{(i)} + \epsilon _d^{(i)})), \end{aligned}$$

where $\epsilon_d^{(i)}\sim\mathcal N(0,\sigma^2)$ and $\sigma$ is fixed to a constant during training, and equals 0 during inference. The sample-specific parameters $\varvec{\mu }^{(i)} \in \mathbb {R}^D, i = 1,...,N$ are predicted based on the gating network $\varvec{\psi }$ such that $\varvec{\mu }^{(i)} = \varvec{\psi }(\varvec{x}^{(i)}|\varvec{\Omega })$, where $\varvec{\Omega }$ are the weights of the gating network. These weights are learned simultaneously with the weights of the prediction network by minimizing the loss in Eq. (5):

5 $$\begin{aligned} \mathbb {E}[\mathcal {L}(\varvec{f_\theta }(\varvec{x}^{(i)} \odot \varvec{z}^{(i)}), y^{(i)}) + \mathcal {R}(\varvec{z}^{(i)})], \end{aligned}$$

where $\mathcal {L}$ is a desired loss (e.g. cross-entropy). $\odot$ represents the Hadamard product (element-wise multiplication), and $\mathcal {R}(\varvec{z}^{(i)})$ is a regularizer term defined in Eq. (6):

6 $$\begin{aligned} \mathcal {R}(\varvec{z}^{(i)}) = \lambda _1||\varvec{z}^{(i)}||_0 + \lambda _2 \sum _j K_{i,j}||\varvec{z}^{(i)} - \varvec{z}^{(j)}||_2^2, \end{aligned}$$

where $K_{i, j} \ge 0$ is a user-defined kernel (e.g., radial basis function). The architecture of the LSPIN model is illustrated in Fig. 8.

## Results

### Classification of Excitatory Vs. Inhibitory Neurons in Humans and Mice

Using the DANN model, we establish that whole-cell current clamp recordings of neurons in mouse brains are similar to those of human brains. Moreover, we show that implementing a joint ANN model to classify cells from human and mouse samples using mutual information from the two domains is possible.

Domain adaptation in low sample size settings is crucial since it enriches data in size and variability. In our example, human neuronal data is more challenging to acquire than data from mice. The significance of our results is that we prove common information is stored in the neuronal data from humans and mice. Since mouse neuronal data is more abundant than neuronal data from human samples, it can be used to strengthen classification models of human neuronal cell types.

**Fig. 9:**
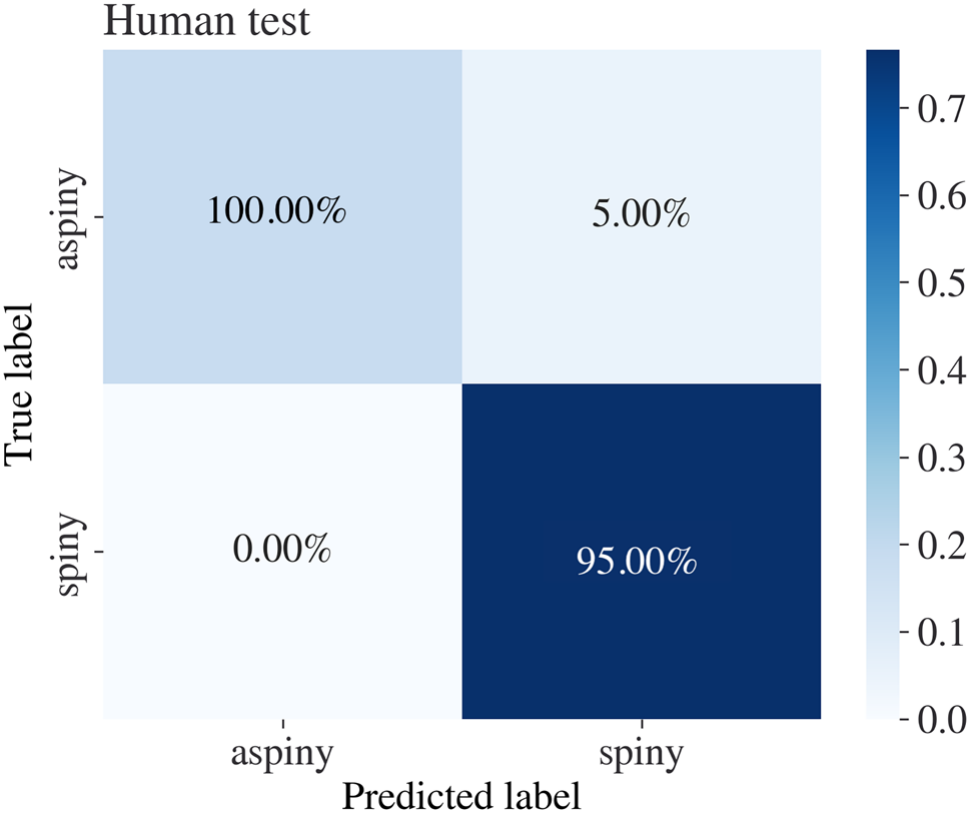
Neuronal cell type classification accuracy percentages, calculated from the total size of the data set in human samples. The vertical axis represents the actual class, while the horizontal axis represents the predicted class

**Fig. 10:**
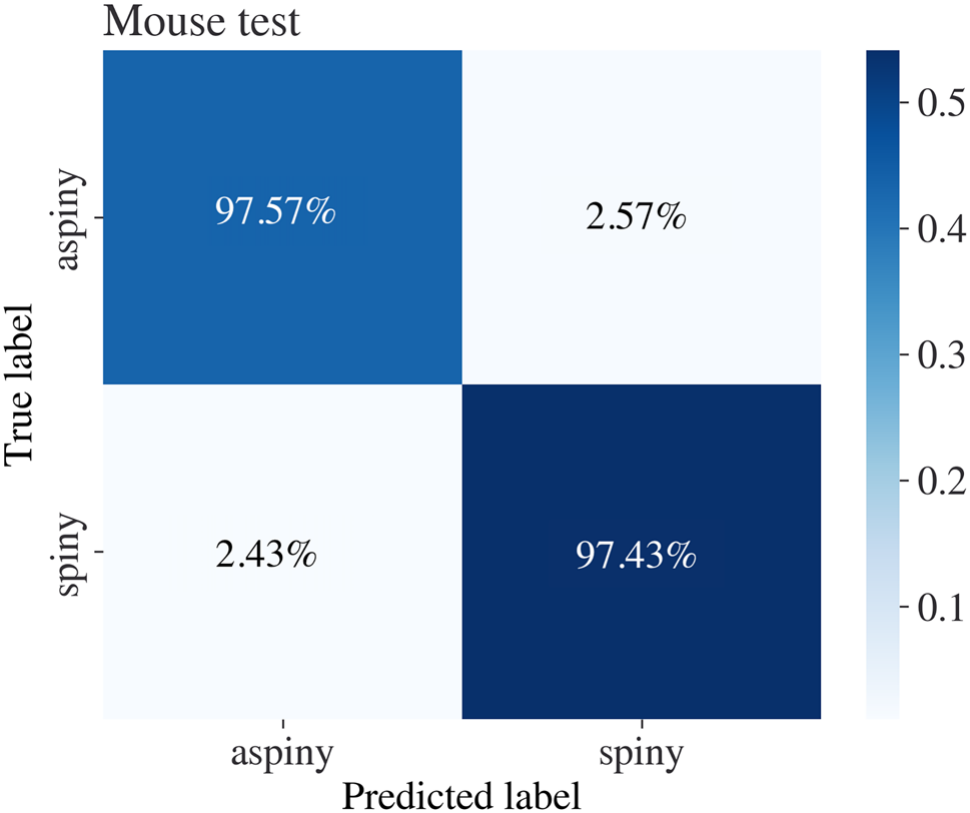
Neuronal cell type classification accuracy percentages, calculated from the total size of the data set in mouse samples. The vertical axis represents the actual class, while the horizontal axis represents the predicted class. The same neural network accurately predicts data from humans and mice simultaneously

We optimized the DANN model on 1378 training samples of cells from both humans and mice. The data was split into 1171 training samples and 207 validation samples, 60 human and 285 mouse cells for testing the model. We used the hyperparameter optimization library, ’Optuna’ Akiba et al. (2019) which enables automated and systematic exploration of the hyperparameter space, to identify the most effective configuration for the model. We used ’Optuna’ over a grid of pre-defined hyperparameters such as the model’s architecture, regularization techniques, learning rates, and optimizers. Each hyperparameter is given a set of different values and ’optuna’ chooses the values which obtain the top metrics based on the validation set. The values that are optimized in this manner are weight decay (chosen between 0.0001 to 1), network architecture (chosen between 6 different architectures including 3 to 4 hidden layers), activation function in each layer (chosen between ’relu’, ’selu’, and ’swish’), learning rate (chosen between 0.001 to 0.2), dropout rate (chosen between 0 to 0.5), batch size (chosen to either be 32 or 64), number of epochs (either 500, 1000, 1500, and 2000), and the optimizer used (chosen to be either ’SGD’ ’RMSProp’ or ’Adam’) The performance of the DANN model is shown in Figs 9, 10 and Table 1. Using the method, we show that the model generalizes to human and mouse domains while providing excellent classification results in accuracy, F1 score, precision, and recall.

The performance of the DANN model was evaluated using the accuracy, F1 score, precision, and recall evaluation metrics. The evaluation shows that the model successfully predicts cell types from humans and mice. Additionally, we compare the DANN model to a FCNN model that was trained and tested on the same dataset, not using an adversarial approach. The FCNN model training process followed the same constraints as the DANN model. The network was optimized using the ’Optuna’ library, following the same constraints as the DANN model. The training process of the DANN model is publicly available at^4^ as well as the training process of the FCNN model at^5^. We demonstrate that optimizing the model using the DANN scheme benefits the classification results in both domains; see Table 1. We demonstrate that the method classifies neurons to their broad types with 95.0% accuracy in human samples and with 97.4% accuracy in mouse samples using the same model weights, delivering a model that generalizes to both the human and mouse domains and classifies samples from both domains simultaneously.

**Table 1:** Comparison of the DANN model optimized using the adversarial approach presented in Section 2.2.1 to two FCNNs not trained using an adversarial scheme for domain adaptation, in terms of accuracy, F1 score, precision and recall. The first FCNN trained on the 41 tabular features presented in the supplementary materials, and the second FCNN trained on the 41 tabular features as well as an organism type feature to allow a complete comparison to the DANN model

**Architecture**	**# Features**	**Domain**	**Evaluation Metric**			
			**Accuracy**	**F1**	**Precision**	**Recall**
FCNN	41	Mouse	0.949	0.955	0.937	0.974
		Human	0.900	0.937	0.957	0.918
FCNN	41 + organism_type	Mouse	0.953	0.959	0.932	0.987
		Human	0.900	0.937	0.957	0.918
**DANN**	**41**	**Mouse**	**0.974**	**0.977**	**0.980**	**0.974**
		**Human**	**0.950**	**0.968**	**1.000**	**0.938**

Ablation: In our evaluation of the model, we discovered that a Fully Connected NN model, trained without using a domain adversarial scheme, and optimized using mouse neuronal data, yet tested on human neuronal data, achieved an accuracy score of 0.646, an F1 score of 0.715, a precision of 0.973, and a recall of 0.565. In contrast, a Fully Connected NN model trained on human neuronal data and tested on mouse neuronal data resulted in an accuracy score of 0.596, F1 score of 0.712, precision of 0.554, and recall of 0.997. This ablation study considers a neural network model with the same architecture of the domain classifier and feature extractor, but with the domain classifier and adversarial scheme removed. The model is then trained on a single source of data, yet tested on the other source of data (trained on human data and tested on mouse data and vice versa). We show that without using an adversarial domain adaptation scheme, results on the domain that the model was not trained on, are lacking. These results demonstrate the domain shift between the two distributions, which the DANN model confounds. This further validates the notion that the DANN model overcomes the domain shift between the two data sources by finding a shared embedding of the two data sources. When classifying samples of neurons from humans and mice, it is important to address the domain shift. Treating the data as a collection of distinct neurons across varied samples leads to lower evaluation metrics compared to using a DANN model, as shown in Table 1. Therefore, it is not feasible to forego domain differentiation and approach the data as a collection varied samples, since the samples are too heterogeneous to be treated as those from a common source.

### Classification of Neurons to Marker Gene Classes in Mice

**Fig. 11:**
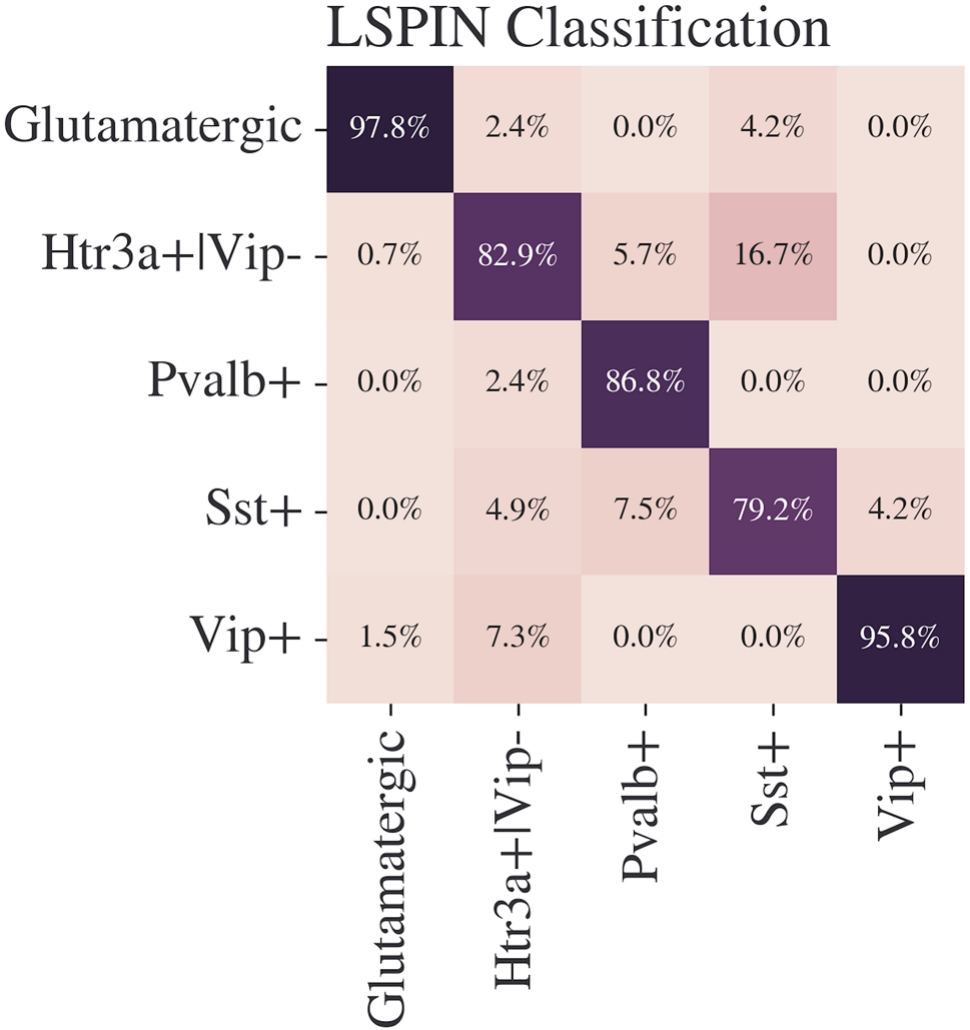
Confusion matrix of the LSPIN model. Accuracy percentages are calculated for each column, which represents the true class, while the horizontal axis represents the predicted class. Glutamatergic neurons are identified 97.8% of the time correctly, *Htr3a*$+|$*Vip*− neurons are identified 82.9% of the time, *Pvalb* 86.8% of the time, *Sst* 79.2% of the time, and *Vip* neurons are identified 95.8% of times correctly. Interestingly, *Sst* neurons are most likely confused with *Htr3a*$+|$*Vip*− neurons, suggesting that the two subtypes have similar electrophysiological properties

Our research proposes the use of the LSPIN model to classify neurons into their five defined subclasses with exceptional accuracy. We achieved an accuracy score of 0.916, an F1 score of 0.877, a precision score of 0.886, and a recall score of 0.873, which surpasses other machine learning models such as RF (Random Forest), SVC (C-Support-Vector-Classifier), and XGBoost (eXtreme Gradient Boosting) in all of the aforementioned metrics. Our results also exceed those of other related studies, as detailed in the introduction. The classification results are presented in Fig. 11 and Table 2.

During the training process, LSPIN was trained on 1105 samples from mice and tested on 277 (42 samples with NaN values from the ACTD were excluded). The prediction network included two hidden dense layers of size 40 and 20 (with an input layer of size 41 and an output layer of size 5). The gating network was assembled from 3 layers, each containing 50 neurons. Tanh was used as the activation function for both the gating and prediction networks. $\lambda _1 = 0.01047, \lambda _2 = 0, \sigma = 0.5$, the network was trained for 1000 epochs with a learning rate of 0.0599 and SGD optimizer.

**Table 2:** Comparison of different machine learning models using macro averaging on the same test data. LSPIN outperforms all other optimized models, achieving an accuracy score of 0.916, an F1 score of 0.877, a precision score of 0.886, and a recall score of 0.873

**Model**	**Evaluation Metric**			
	**Accuracy**	**F1**	**Precision**	**Recall**
RF	0.824	0.726	0.774	0.702
SVC	0.837	0.749	0.789	0.731
XGBoost	0.850	0.768	0.779	0.759
**LSPIN**	**0.916**	**0.877**	**0.886**	**0.873**

**Fig. 12:**
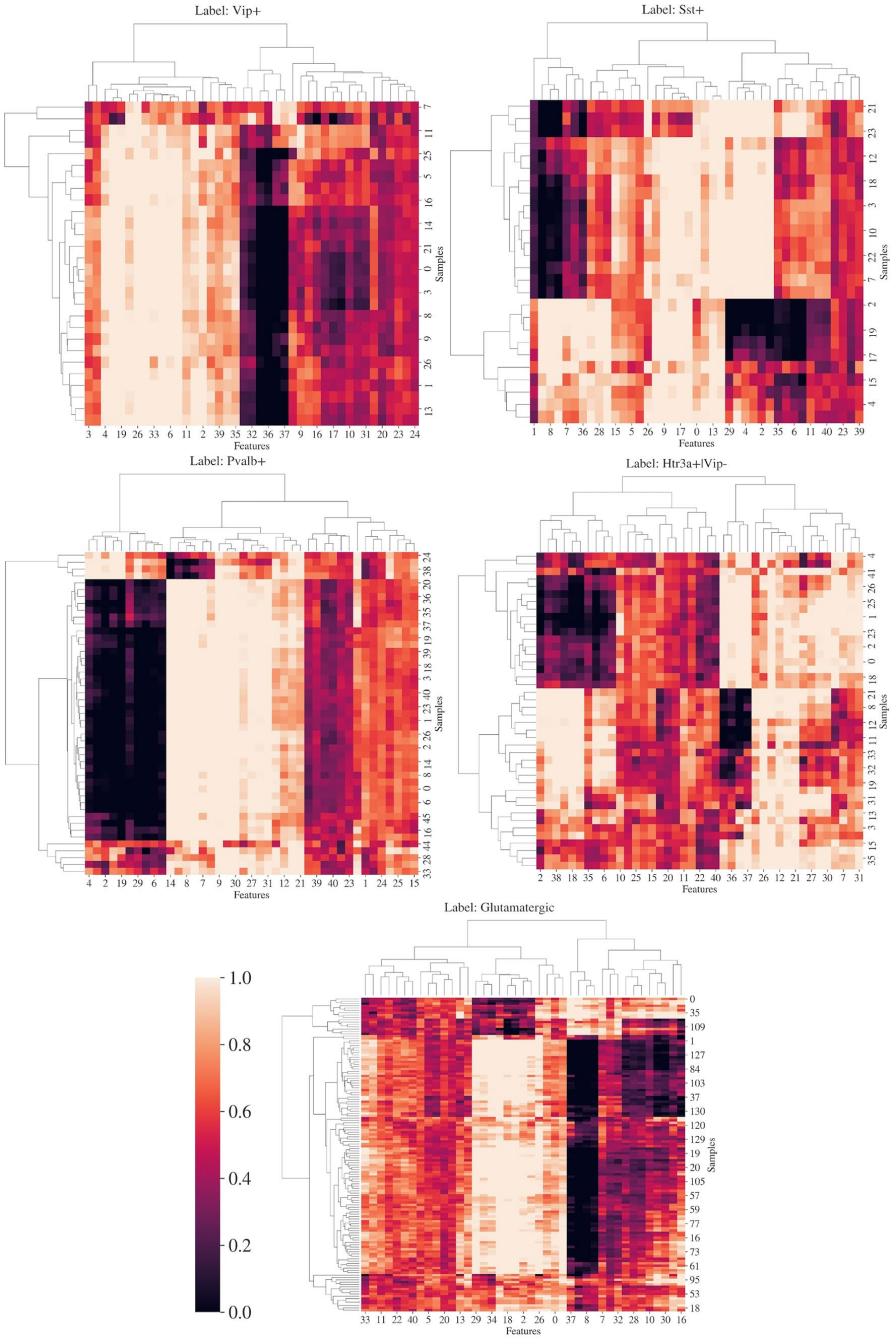
Hierarchical clustering of the gate matrix. Features are multiplied by a constant expressed as the color of the cell according to the color bar present. Features that are completely muted are displayed in black, while features that stay unchanged are displayed in white. The vertical axis represents the test samples, and the horizontal axis represents the electrophysiological features

We can interpret the decisions produced by the prediction network via the gating network and stochastic gates outputs. The model is forced to select a subset of the most informative features identified for each sample; by that, the feature space is diminished, and model overfitting is reduced in low-sample-size data. The selection of relevant features produces an interpretable classification verdict in which relevant and non-relevant features are identified for each neuronal subclass, less essential features are attenuated, while important features are kept. See Fig. 12.

## Discussion

The paper introduces two classification frameworks for identifying neuronal types based on electrophysiological recordings. The first method enriches mouse data by considering shared information from mouse and human neuronal samples. To overcome the domain shift between the mouse and human distributions, we use DANN. The joint model accurately classifies cells to their broad type with 95.0% accuracy for human cells and 97.4% accuracy for mouse cells.

The second method uses LSPIN, a locally sparse ANN, to identify five neuronal subtypes. The method overcomes low sample-size data by automatically subsetting the feature space and excluding non-essential features. Additionally, it is inherently interpretable, revealing the more or less significant features for the classification. In our case, it provides biological insights into the defining features of each neuronal class.

It is also possible to combine the two methods by modifying the DANN architecture to have the LSPIN’s gating network as the DANN’s feature extractor, so that the DANN’s feature extractor provides local sparsification of features.

There are several avenues for future research in the field of neuronal classification. Firstly, expanding the range of species included in the classification process could provide valuable insights into the evolutionary conservation of different types of neurons and their functional properties. The DANN method is capable of handling domain adaptation from multiple sources, making it suitable for this type of research. Secondly, utilizing the DANN method in an unsupervised scheme could improve the accuracy of interneuron subcategory predictions for human neuronal samples. Thirdly, the generalization ability of the models should be investigated to determine their applicability in real-world scenarios. This can be achieved by evaluating the framework’s performance on independent datasets from different laboratories and experimental conditions. Finally, exploring the biological insights of each neuronal type would enhance the interpretability of the model’s predictions. This would provide a better understanding of the specific features and regions of the electrophysiological signals that contribute most significantly to the classification and characterization of different neuronal types.

## Information Sharing Statement

Data sharing is not applicable to this article as no new data were created in this study. Data used in the preparation of this article are publicly available at https://celltypes.brain-map.org/data (ACTD). The AllenSDK software library is open sourced and available at https://allensdk.readthedocs.io/en/latest/, the source code of the research is also publicly available at https://github.com/ofek181/Neuronal-Cell-Type-Classification.

## Supplementary Information

Below is the link to the electronic supplementary material.

Supplementary file1 (DOCX 92 KB)

## Data Availability

All data used in the research is from the Allen Cell Types Database, found at: https://celltypes.brain-map.org/data.

## Footnotes

1 https://github.com/ofek181/Neuronal-Cell-Type-Classification

2 https://celltypes.brain-map.org/data

3 http://help.brain-map.org/display/celltypes/Documentation

4 https://github.com/ofek181/Neuronal-Cell-Type-Classification/blob/master/dann_dendrite_type.py

5 https://github.com/ofek181/Neuronal-Cell-Type-Classification/blob/master/domain_adaptation_comparison.py
