# Supplementary material for: Classifying Neuronal Cell Types Based on Shared Electrophysiological Information from Humans and Mice
Source: Neuroinformatics. 2024 Jul 8;22(4):473–86. doi: 10.1007/s12021-024-09675-5 (PMC11579117; doi:10.1007/s12021-024-09675-5)
Supplement: Supplementary file 1 — Supplementary file1 (DOCX 92 KB) [file 12021_2024_9675_MOESM1_ESM.pdf]

#	Feature	#	Feature	#	Feature
1	threshold_v_noise	16	fast_trough_v_long_square	31	threshold_v_long_square
2	threshold_i_noise	17	fast_trough_v_ramp	32	threshold_v_ramp
3	peak_v_noise	18	fast_trough_v_short_square	33	threshold_v_short_square
4	peak_i_noise	19	input_resistance_mohm	34	trough_v_long_square
5	trough_v_noise	20	latency	35	trough_v_ramp
6	trough_i_noise	21	peak_v_long_square	36	trough_v_short_square
7	upstroke_noise	22	peak_v_ramp	37	upstroke_downstroke_ratio_long_square
8	upstroke_v_noise	23	peak_v_short_square	38	upstroke_downstroke_ratio_ramp
9	downstroke_noise	24	ri	39	upstroke_downstroke_ratio_short_square
10	downstroke_v_noise	25	sag	40	vm_for_sag
11	width	26	seal_gohm	41	vrest

Supplementary information. A full description of each feature and its meaning is found at the Ephys white paper of the Allen Cell Type database at: Allen Cell Types documentation.
